# Supplementary material for: Seminoma and Embryonal Carcinoma Footprints Identified by Analysis of Integrated Genome-Wide Epigenetic and Expression Profiles of Germ Cell Cancer Cell Lines
Source: PLoS One. 2014 Jun 2;9(6):e98330. doi: 10.1371/journal.pone.0098330 (PMC4041891; doi:10.1371/journal.pone.0098330)
Supplement: File S1 — ZIP file containing DMRforPairs output for significant regions. Please start from the html files. (ZIP) [file pone.0098330.s008.zip › figures/42135.pdf]

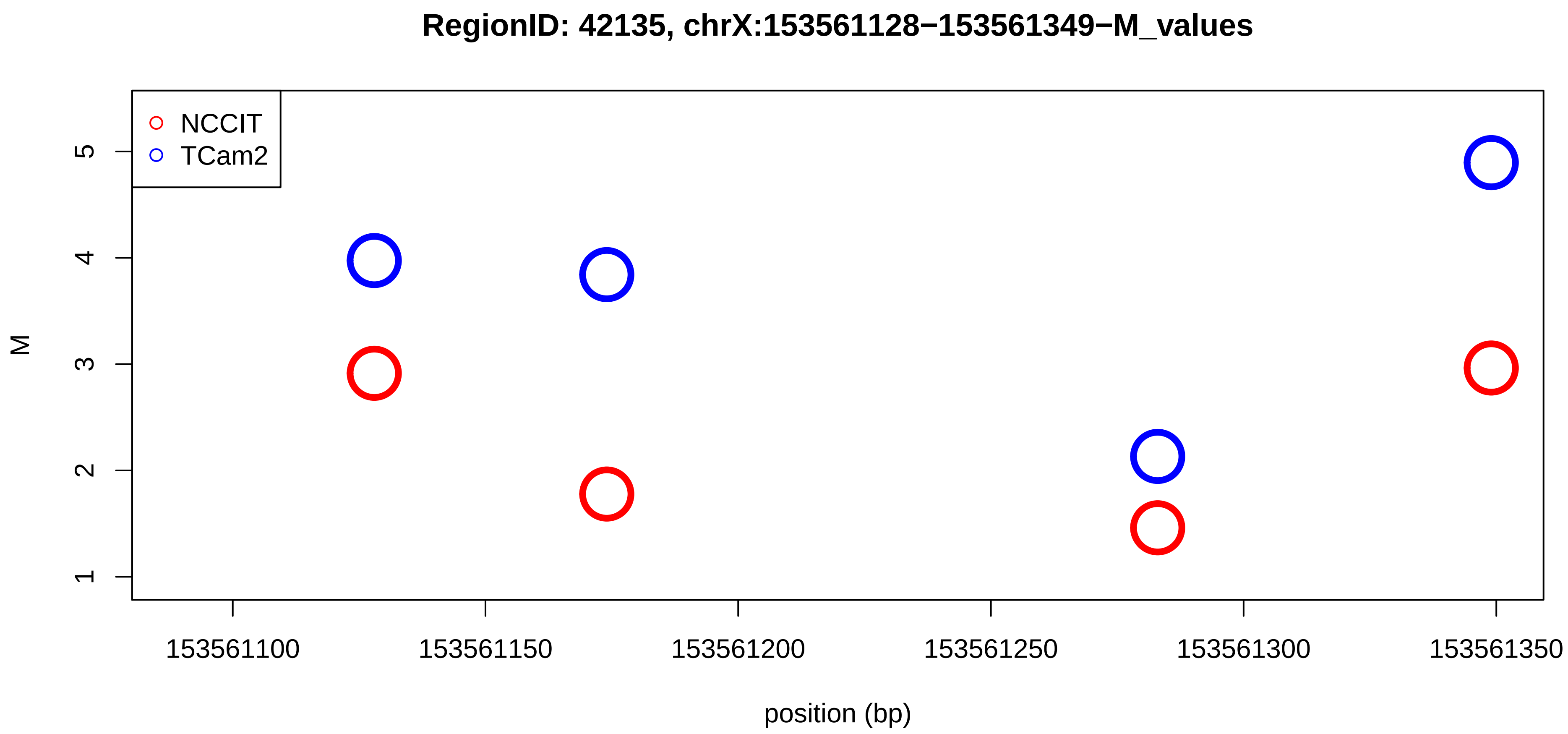

RegionID: 42135, chrX:153561128−153561349−M_values
NCCIT
TCam2
M
1
2
3
4
5
153561100
153561150
153561200
153561250
153561300
153561350
position (bp)

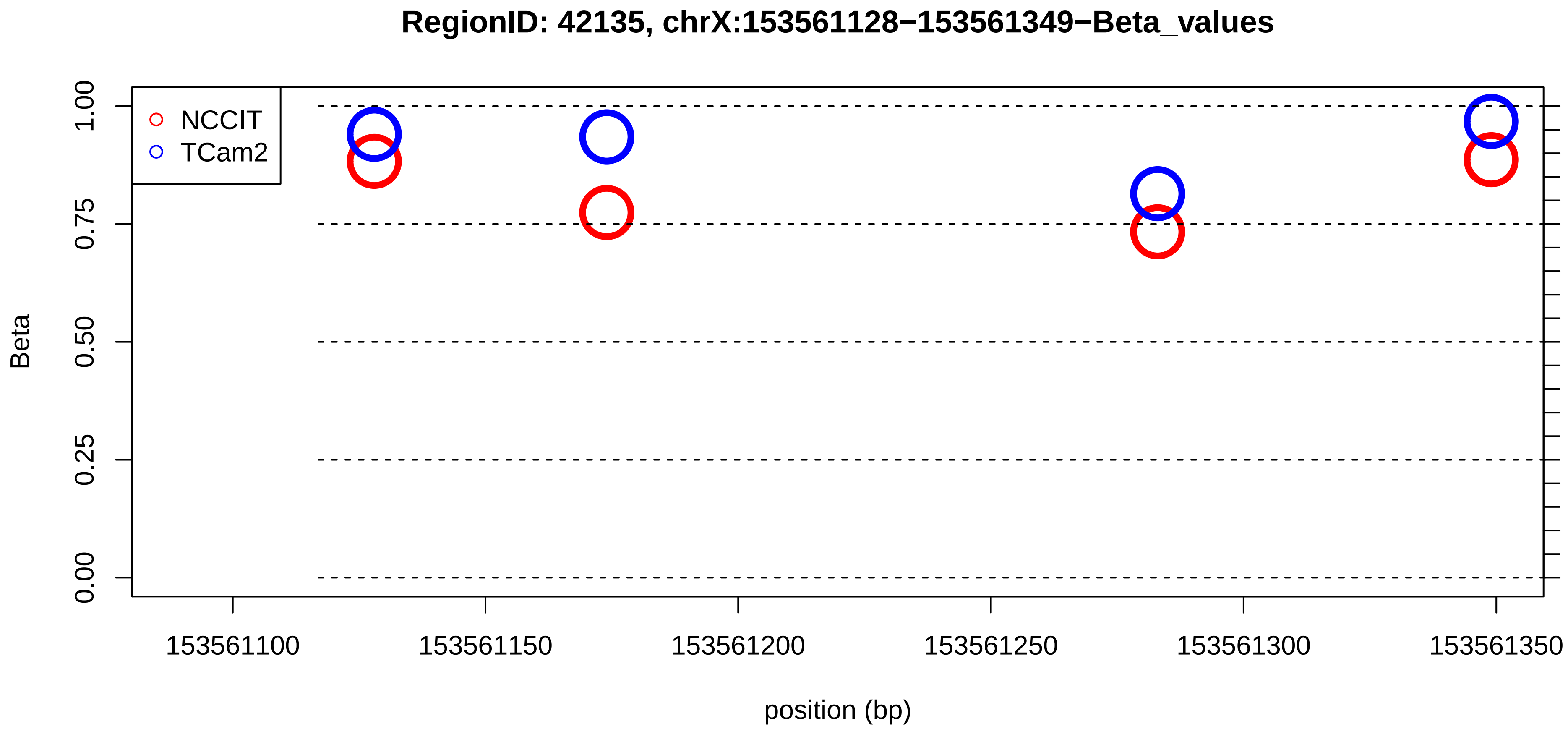

RegionID: 42135, chrX:153561128−153561349−Beta_values
NCCIT
TCam2
Beta
0.00
0.25
0.50
0.75
1.00
153561100
153561150
153561200
153561250
153561300
153561350
position (bp)
